# Factors associated with unfavorable outcomes in patients with acute abdominal pain visiting the emergency department

**DOI:** 10.1186/s12873-022-00761-y

**Published:** 2022-12-06

**Authors:** Ar-aishah Dadeh

**Affiliations:** Department of Emergency Medicine, Faculty of Medicine, Songklanagarind Hospital, Prince of Songkla University, Hat Yai, Songkhla, 90110 Thailand

**Keywords:** Acute abdominal pain, Emergency department, Unfavorable outcome, Invasive procedure

## Abstract

**Background:**

Unfavorable outcomes occur in patients with acute abdominal pain who visit the emergency department (ED). We aimed to determine the factors associated with unfavorable outcomes in patients with acute abdominal pain visiting the ED.

**Methods:**

This retrospective cohort study was conducted from July 1, 2015 to June 30, 2016. The inclusion criterion was patients aged older than 18 years who presented to the ED with acute abdominal pain. Significant factors associated with unfavorable outcomes were examined using univariate and multivariate logistic regression analyses.

**Results:**

A total of 951 patients were included in the study. Multivariate logistic regression analysis showed that the ED length of stay (EDLOS) > 4 h (adjusted odds ratio (AOR) 2.62, 95% confidence interval [CI]: 1.33–5.14; *p* = 0.005), diastolic blood pressure (DBP) < 80 mmHg (AOR 3.31, 95% CI: 1.71–6.4; *p* ≤ 0.001), respiratory rate ≥ 24 breaths/min (AOR 2.03, 95% CI: 1.07–3.86; *p* ≤ 0.031), right lower quadrant (RLQ) tenderness (AOR 3.72, 95% CI: 1.89–7.32; *p* ≤ 0.001), abdominal distension (AOR 2.91, 95% CI: 1.29–6.57; *p* = 0.010), hypoactive bowel sounds (AOR 2.89, 95% CI: 1.09–7.67; *p* = 0.033), presence of specific abdominal signs (AOR 2.07, 95% CI: 1.1–3.88; *p* = 0.024), white blood cell count ≥ 12,000 cells/mm^3^ (AOR 2.37, 95% CI: 1.22–4.6; *p* = 0.011), and absolute neutrophil count (ANC) > 75% (AOR 2.83, 95% CI: 1.39–5.75; *p* = 0.004) were revealed as significant factors associated with unfavorable outcomes.

**Conclusions:**

The present study revealed that the significant clinical signs associated with the occurrence of unfavorable outcomes were DBP < 80 mmHg, tachypnea (≥ 24 breaths/min), RLQ tenderness, abdominal distension, hypoactive bowel sounds, and presence of specific abdominal signs. Moreover, the associated laboratory results identified in this study were leukocytosis and ANC > 75%. Additionally, patients with abdominal pain visiting the ED who had an EDLOS longer than 4 h were associated with unfavorable outcomes.

**Supplementary Information:**

The online version contains supplementary material available at 10.1186/s12873-022-00761-y.

## Background

Acute abdominal pain is a common presenting symptom in the emergency department (ED) accounting for 5–10% of all ED visits [1]. Sometimes the symptoms presented in the ED lead to serious adverse outcomes, particularly in patients with acute abdominal pain. Several modifying factors, such as underlying diseases, immune status, and inability to communicate, can cause a more complicated disease process [2]. The skills of healthcare providers along with effective diagnostic tools are important factors that can lead to an accurate diagnosis and management. Although many advanced diagnostic and therapeutic tools are available, the misdiagnosis rate of acute abdominal pain has changed little over time [2]. A missed diagnosis leads to increased morbidity and mortality. Serious abdominal pathologies that are frequently misdiagnosed include gastroenteritis, gastritis, urinary tract infection, pelvic inflammatory infection, and constipation. Life-threatening conditions sometimes missed in the ED include ruptured abdominal aortic aneurysms, appendicitis, ectopic pregnancy, diverticulitis, a perforated viscus, mesenteric ischemia, and bowel obstruction [1]. Patients who present early and receive immediate corrective measures have better outcomes. However, patients who present late experience unfavorable outcomes, with significantly more complications. Therefore, early detection and treatment of acute abdominal pain are essential [3].

Vital signs are commonly used among triage systems, along with risk scoring tools in the ED [4]. The type and number of abnormal vital signs are strong predictors for in-hospital mortality and admission to the intensive care unit (ICU) [5]. The presentation of a patient with abdominal pain and abnormal vital signs may indicate a serious surgical diagnosis, such as a ruptured abdominal aortic aneurysm, serious intra-abdominal infection, or ruptured hollow viscus organ [6].

Endotracheal intubation and central venous catheter (CVC) placement are common invasive procedures performed in critically ill patients in the ED. Patients who require invasive procedures and are admitted to the ICU from the ED have an in-hospital mortality rate of 21.9% [7]. The reported complications of emergency endotracheal intubation in the ED include hemodynamic collapse (9%), aspiration (4.5%), emergent tracheostomy (1.1%), and pneumothorax (0.6%) [8]. The risks associated with CVC placement range from clinically insignificant to fatal. These risks include bleeding, pneumothorax, nerve injury, and severe arrhythmia [9].

The overall mortality rates of patients in the ED who present with abdominal pain at 24 h and 7 days are 2% and 4%, respectively, whereas the overall in-hospital mortality rate is 8%. The risk factors for mortality are male sex, hypoglycemia, ICU admission, receipt of intravenous fluids, and need for surgery [10].

To the best of our knowledge, factors that affect unfavorable events or outcomes have not been explored. This study aimed to determine the factors associated with unfavorable outcomes in patients with acute abdominal pain visiting the ED.

## Methods

### Study design and setting

This retrospective cohort study was conducted in the ED of a teaching hospital and a tertiary care medical center with a capacity of 850 beds. The ED has more than 48,000 patient visits per year. The data were collected from July 1, 2015 to June 30, 2016. The inclusion criterion was patients aged older than 18 years with acute abdominal pain visiting the ED. The exclusion criteria were patients scheduled for elective surgery, having undergone previous abdominal surgery within the previous 6 months, with prior diagnosis of cancer, with chronic abdominal pain, with traumatic abdominal pain, who were pregnant, and with incomplete medical records. A total of 951 patients were included in the retrospective study.

### Operational definitions

Acute abdominal pain was defined as an abrupt onset of pain or soreness that appeared within 7 days before presenting to the ED and necessitated prompt diagnosis and aggressive treatment, typically a surgical intervention [10, 11]. Unfavorable outcomes were defined as the occurrence of one or more of the following: (i) shock that required an invasive procedure during the ED stay (e.g., CVC insertion and mechanical ventilation), (ii) emergency surgery, (iii) presence of post-operative complications, and (iv) occurrence of in-hospital cardiac arrest (IHCA) in the ED or after admission [3, 12].

### Data collection

The data collected from the electronic medical records and ED data registry included baseline characteristics, triage category, physical examination findings, symptom duration, laboratory and imaging results, final diagnosis, treatment in the ED, ED length of stay (EDLOS), ED disposition, morbidity, and mortality. Patients were divided into either an unfavorable outcome group or a favorable outcome group.

### Outcome measurements

The primary outcomes were the factors associated with unfavorable outcomes in patients with acute abdominal pain visiting the ED. The secondary outcome was the association between the factors affecting unfavorable outcomes and mortality.

### Statistical analyses

The n4Studies tool was used to determine the sample size of the study population to evaluate two independent proportions. The final calculated sample size was 865 patients. After adjusting for a 10% dropout rate, the desired sample size was 952. R software version 4.0.2 was used to perform statistical analyses after all data were imported into EpiData version 3.1 (R Foundation, Vienna, Austria). Continuous variables were analyzed and reported as means and medians whereas categorical variables are reported as percentages. Student’s t-test was used for continuous and ordinal variables and Pearson’s chi-squared test was used for categorical variables. A multivariate logistic regression model was used to evaluate the factors associated with unfavorable outcomes. We determined the factors associated with unfavorable outcomes using backward stepwise logistic regression. Significant factors associated with unfavorable outcomes (*p* < 0.2) identified during univariate logistic regression analysis were introduced into a subsequent multivariate logistic regression analysis. The optimal clinical cut-off points to predict unfavorable outcome were determined based on the best sensitivity and specificity results. The accuracy of clinical signs and laboratory results of the associated factors to predict unfavorable outcomes was determined using receiver operating characteristic (ROC) curves and area under the ROC curve (AUROC). Model discrimination was rated as good if it produced an AUROC between 0.7 and 0.8 and excellent if it produced an AUROC between 0.8 and 0.9 [13]. Analytical results were described as odds ratio (OR) with 95% confidence interval (CI). Statistical significance was set at *p*-value < 0.05.

### Compliance with ethical requirements

This study was approved by the Ethics Committee of Prince of Songkla University (approval number: REC 58–158-20–1). The Institutional Review Board of Prince of Songkla University is affiliated with the International Conference on Harmonization in Good Clinical Practice. The requirement for informed consent was waived in accordance with our institutional review board's policy because the participants had no greater than minimum risk and the patients received standard medical care. All research information was kept confidential in an encrypted file with a password and limited data access by only the researcher and assistant. This study was conducted in accordance with the principles of Declaration of Helsinki.

## Results

### Demographic data

A total of 3,784 patients with abdominal discomfort as their primary complaint were registered during the study period. Using a computer-based randomization approach, 951 patients who met the inclusion criterion were selected. Of these, 351 patients (36.9%) were men and 600 patients (63.1%) were women. The baseline characteristics of the unfavorable outcome and favorable outcome groups are shown in Table 1. The median age (interquartile range, IQR) of the unfavorable outcome group was older than the favorable outcome group (51 [29, 65.5] vs. 42 [28, 60]) without statistical significance. Upon comparing the two groups, the statistically significant factors were underlying gastrointestinal disease, triage level, EDLOS, hospital LOS, admission to ward, admission to ICU, discharge from ED, hospital discharge status whether survived or dead, need for consultation, and having consulted specialty.

**Table 1:** Clinical factors and baseline characteristics of patients with acute abdominal pain who presented at the ED

Factors	Unfavorable outcomes (*n* = 75)	Favorable outcomes (*n* = 876)	Total (*n* = 951)	*p*-value
**Age, y, median (IQR)**	51 (29,65.5)	42 (28,60)	43 (28,61)	0.134
**Sex**				0.051
Male	36 (48)	315 (36)	351 (36.9)	
Female	39 (52)	561 (64)	600 (63.1)	
**Comorbidities**				0.122
Present	44 (58.7)	426 (48.6)	470 (49.4)	
Absent	31 (41.3)	450 (51.4)	481 (50.6)	
Hypertension	14 (18.7)	137 (15.6)	151 (15.9)	0.600
Gastrointestinal disease	14 (18.7)	90 (10.3)	104 (10.9)	0.041
Diabetes mellitus	8 (10.7)	64 (7.3)	72 (7.6)	0.407
Cardiovascular disease	2 (2.7)	35 (4)	37 (3.9)	0.762
Chronic heart disease	2 (2.7)	4 (0.5)	6 (0.6)	0.075
Chronic pulmonary disease	2 (2.7)	19 (2.2)	21 (2.2)	0.678
Cerebrovascular disease	2 (2.7)	27 (3.1)	29 (3)	1.000
Chronic renal failure	5 (6.7)	25 (2.9)	30 (3.2)	0.080
Others	28 (37.3)	284 (32.4)	312 (32.8)	0.458
**History of abdominal surgery**				0.957
Yes	9 (12)	97 (11.1)	106 (11.1)	
No	66 (88)	779 (88.9)	845 (88.9)	
**Triage levels**				< 0.001
ESI 1	0	0	0	
ESI 2	12 (16)	32 (3.7)	44 (4.6)	
ESI 3	48 (64)	411 (46.9)	459 (48.3)	
ESI 4	14 (18.7)	402 (45.9)	416 (43.7)	
ESI 5	1 (1.3)	31 (3.5)	32 (3.4)	
**Time from onset of symptoms to ED visit (h), median (IQR)**	22 (6.5–24)	10 (4,39)	12 (4–30)	0.145
**EDLOS (h), median (IQR)**	5.25 (4.3–6.6)	2.55 (1.68–3.7)	2.6 (1.75–4.05)	< 0.001
**Hospital LOS (days), median (IQR)**	4.4 (2.5–7.1)	3.1 (1.2–5.1)	3.8 (2–6)	0.004
**ED dispositions**				
EDOU	1 (1.3)	14 (1.6)	15 (1.6)	1.000
Admission to ward	64 (85.3)	46 (5.3)	110 (11.6)	< 0.001
Admission to ICU	7 (9.3)	0 (0)	7 (0.7)	< 0.001
Discharge	0 (0)	812 (92.7)	812 (85.4)	< 0.001
Referred	2 (2.7)	5 (0.6)	7 (0.7)	0.099
Dead at the ED	1 (1.3)	0 (0)	1 (0.1)	0.079
**Hospital discharge status**				< 0.001
Survived	71 (94.7)	876 (100)	947 (99.6)	
Dead	4 (5.3)	0 (0)	4 (0.4)	
**Consultation needed**	75 (100)	77 (8.8)	152 (16)	< 0.001
**Specialty**				0.042
Internal medicine	8 (16.3)	11 (19.6)	19 (18.1)	
Surgical	35 (71.4)	27 (48.2)	62 (59)	
Gynecological	6 (12.2)	17 (30.4)	23 (21.9)	
Psychiatry	0 (0)	1 (1.8)	1 (1)	

Data are presented as *n* (%), unless otherwise indicated

*IQR* interquartile range, *ESI* emergency severity index, *ED* emergency department, *EDLOS* emergency department length of stay, *LOS* length of stay, *EDOU* emergency department observation unit, *ICU* intensive care unit

Five diagnoses caused acute abdominal pain in all patients: dyspepsia (23.97%, 228 patients), gastroenteritis (14.93%, 142 patients), ureteric stones (13.88%, 132 patients), urinary tract infection (7.36%, 70 patients), and gynecological conditions (5.99%, 57 patients). The three main diagnoses in the unfavorable outcome group were acute appendicitis (48%, 36 patients), intestinal obstruction (14.66%, 11 patients), and gynecological conditions which included a ruptured ectopic pregnancy (6.66%, 5 patients) and pelvic inflammatory disease (2.66%, 2 patients) (Table 2).

**Table 2:** Diagnoses and causes of acute abdominal pain in the study population

	**Unfavorable outcomes (*n* = 75)**	**Favorable outcomes (*n* = 876)**	**Total** **(*n* = 951)**	***p*-value**
**Emergency department diagnosis**				< 0.001
Cause identified	75 (100)	753 (86)	828 (87.1)	
Nonspecific abdominal pain	0 (0)	123 (14)	123 (12.9)	
**Gastrointestinal conditions**	67 (89.3)	517 (59)	584 (61.4)	< 0.001
Appendicitis	36 (53.7)	11 (2.1)	47 (8)	< 0.001
Gallstones	2 (3)	22 (4.3)	24 (4.1)	1.000
Diverticulitis	3 (4.5)	7 (1.4)	10 (1.7)	0.096
Constipation	1 (1.5)	31 (6)	32 (5.5)	0.159
Hollow viscus organ perforation	4 (6)	0 (0)	4 (0.7)	< 0.001
Cholecystitis	2 (3)	7 (1.4)	9 (1.5)	0.276
Pancreatitis	4 (6)	5 (1)	9 (1.5)	0.013
Intestinal obstruction	11 (16.4)	13 (2.5)	24 (4.1)	< 0.001
Gastroenteritis	0 (0)	142 (27.5)	142 (24.3)	< 0.001
Dyspepsia	1 (1.5)	227 (43.9)	228 (39)	< 0.001
Peptic ulcer	0 (0)	2 (0.4)	2 (0.3)	1.000
Aortic aneurysm	1 (1.5)	0 (0)	1 (0.2)	0.115
Colonic obstruction	1 (1.5)	1 (0.2)	2 (0.3)	0.216
Cholelithiasis/biliary tract disease	3 (4.5)	11 (2.1)	14 (2.4)	0.21
Others	9 (13.4)	72 (13.9)	81 (13.9)	1.000
**Genitourinary conditions**	3 (4)	189 (21.6)	192 (20.2)	< 0.001
Ureteric stone	1 (33.3)	131 (69.3)	132 (68.8)	0.231
Urinary tract infection	1 (33.3)	69 (36.5)	70 (36.5)	1.000
Other genitourinary conditions	1 (33.3)	5 (2.6)	6 (3.1)	0.091
**Gynecological conditions**	7 (9.3)	50 (5.7)	57 (6)	0.203
Ectopic pregnancy with complication	5 (71.4)	0 (0)	5 (8.8)	< 0.001
Ectopic pregnancy without complication	0 (0)	1 (2)	1 (1.8)	1.000
Ovarian disease with complication	0 (0)	5 (10)	5 (8.8)	1.000
Ovarian disease without complication	0 (0)	1 (2)	1 (1.8)	1.000
Pelvic inflammatory disease	2 (28.6)	7 (14)	9 (15.8)	0.304
Tubo-ovarian abscess	0 (0)	1 (2)	1 (1.8)	1.000
Other gynecological conditions	0 (0)	36 (72)	36 (63.2)	< 0.001
**Extra-abdominal conditions**	0 (0)	15 (1.7)	15 (1.6)	0.623

Data are presented as *n* (%)

### Factors affecting the occurrence of unfavorable outcomes

An EDLOS longer than 4 h was highly associated with predicting the occurrence of unfavorable outcome with an AUROC of 0.783, and EDLOS had a high negative predictive value of 98%. The study period also had 77% sensitivity and 79% specificity for predicting unfavorable outcomes (Table 3). The univariate logistic regression analysis showed that the presence of comorbidities of chronic heart disease (OR 5.97, 95% CI: 1.08–33.16), emergency severity index triage level 2 (OR 11.62, 95% CI: 1.43–94.83), and EDLOS longer than 4 h (OR 13.1, 95% CI: 7.45–23.04) increased the likelihood of unfavorable outcomes. Furthermore, the following physical examination findings also increased the likelihood of unfavorable outcome: presence of hypotension (systolic blood pressure [< 100 mmHg]) (OR 3.28, 95% CI: 0.89–12.01), diastolic blood pressure (DBP) < 80 mmHg (OR 3.01, 95% CI: 1.78–5.11, tachycardia (heart rate > 90 bpm) (OR 2.39, 95% CI:1.49–3.85), tachypnea (respiratory rate [RR] ≥ 30 breaths/min) (OR 5.02, 95% CI: 2.4–10.51), and fever (body temperature ≥ 38 °C) (OR 6.58, 95% CI: 3.31–13.11). Moreover, the abdominal signs identified on univariate logistic regression analysis with high ORs were generalized abdominal rigidity/guarding (OR 39.68, 95% CI: 10.49–150.1), localized rigidity/guarding (OR 22.31, 95% CI: 7.86–63.3), right lower quadrant (RLQ) tenderness (OR 6.9, 95% CI: 3.83–10.33), and hypoactive bowel sounds (OR 6.26, 95% CI: 3.16–12.38). An absolute neutrophil count (ANC) > 75% (OR 4.4, 95% CI: 2.44–7.93), white blood cell (WBC) count ≥ 12,000 cells/mm^3^ (OR 3.39, 95% CI: 2–5.74), and lymphopenia < 15% (OR 3.4, 95% CI:1.95–5.95) were also associated with unfavorable outcomes (Table 4). However, EDLOS > 4 h, DBP < 80 mmHg, RR > 24 breaths/min, RLQ tenderness, abdominal distension, hypoactive bowel sounds, presence of specific abdominal signs (i.e., Murphy’s sign, Rovsing’s sign, psoas sign), WBC count ≥ 12,000 cells/mm^3^, and ANC > 75% were revealed to be significant factors in the multivariate logistic regression analysis, with statistical significance (Table 5).

**Table 3:** Accuracy of vital signs, abdominal signs, and laboratory results in predicting unfavorable outcomes in patients with acute abdominal pain

Variables	AUROC	Sensitivity	Specificity	LR + (95% CI)	LR − (95% CI)	PPV	NPV
**Age ≥ 65 years**	0.5530594	0.29	0.81	1.57	0.87	0.12	0.93
**Vital signs**							
SBP < 125 mmHg	0.5605708	0.45	0.67	1.36	0.82	0.10	0.93
SBP < 100 mmHg	0.5137215	0.04	0.99	3.19	0.97	0.21	0.92
DBP < 80 mmHg	0.6280822	0.73	0.52	1.54	0.51	0.12	0.96
RR ≥ 24 breaths/min	0.5650228	0.59	0.54	1.28	0.76	0.10	0.94
**Abdominal tenderness**							
Generalized	0.6722063	0.47	0.88	3.82	0.61	0.25	0.95
Epigastrium	0.5709132	0.24	0.90	2.44	0.84	0.17	0.93
RLQ	0.5756393	0.19	0.96	5.27	0.84	0.31	0.93
RUQ	0.610137	0.63	0.59	1.54	0.63	0.12	0.95
**Laboratory results**							
WBC count ≥ 12,000	0.6469595	0.61	0.69	1.94	0.57	0.33	0.88
ANC > 75%	0.6689189	0.77	0.57	1.78	0.40	0.31	0.91
**EDLOS > 4 h**	0.7833562	0.77	0.79	3.74	0.29	0.24	0.98

*AUROC* area under receiver operating characteristic curve, *LR* + positive likelihood ratio, *LR − *negative likelihood ratio, *PPV* positive predictive value, *NPV* negative predictive value, *CI* confidence interval, *SBP* systolic blood pressure, *DBP* diastolic blood pressure, *RR* respiratory rate, *RLQ* right lower quadrant, *RUQ* right upper quadrant, *WBC* white blood cell, *ANC* absolute neutrophil count, *EDLOS* emergency department length of stay

**Table 4:** Univariate logistic regression analysis of factors that affected unfavorable outcomes

Variables	Odds ratio	95% CI	*p-*value
Sex: male vs. female	1.64	1.02–2.64	0.04
Age ≥ 65 y	1.8	1.07–3.05	0.028
Presence of comorbidities	1.5	0.93–2.42	0.097
Chronic heart disease	5.97	1.08–33.16	0.041
Chronic renal failure	2.43	0.9–6.55	0.079
Gastrointestinal disease	2.0	1.08–3.73	0.028
Triage level			
ESI 2	11.62	1.43–94.83	0.022
ESI 3	3.62	0.48–27.12	0.21
ESI 4	1.08	0.14–8.48	0.942
Onset of symptoms to ED visit > 12 h	1.73	1.07–2.8	0.026
EDLOS > 4 h	13.1	7.45–23.04	< 0.001
Hospital length of stay > 7 days	2.17	0.87–5.41	0.098
**Vital signs**			
SBP < 125 mmHg	1.67	1.04–2.68	0.035
SBP < 100 mmHg	3.28	0.89–12.01	0.073
DBP < 80 mmHg	3.01	1.78–5.11	< 0.001
Heart rate ≥ 90 bpm	2.39	1.49–3.85	< 0.001
RR ≥ 24 breaths/min	1.69	1.05–2.73	0.032
RR ≥ 30 breaths/min	5.02	2.4–10.51	< 0.001
BT ≥ 37.5 °C	5.02	3.04–8.29	< 0.001
BT ≥ 38 °C	6.58	3.31–13.11	< 0.001
**Abdominal tenderness**			
Generalized	2.3	1.18–4.48	0.014
Epigastrium	0.36	0.17–0.77	0.008
RLQ	6.9	3.83–10.33	< 0.001
RUQ	2.04	0.96–4.3	0.063
Abdominal distension	2.9	1.63–5.15	< 0.001
Generalized rigidity/guarding	39.68	10.49,150.1	< 0.001
Localized rigidity/guarding	22.31	7.86–63.3	< 0.001
Hypoactive bowel sounds	6.26	3.16–12.38	< 0.001
Presence of specific abdominal signs	2.45	1.51–3.99	< 0.001
Digital rectal examination	3.3	1.74–6.25	< 0.001
**Laboratory results**			
White blood cell count ≥ 12,000	3.39	2–5.74	< 0.001
White blood cell count ≥ 14,000	3.19	1.86–5.5	< 0.001
ANC > 75%	4.4	2.44–7.93	< 0.001
Lymphocytes < 15%	3.4	1.95–5.95	< 0.001
Positive urinalysis	0.3	0.15–0.59	< 0.001

*CI* confidence interval, *ESI* emergency severity index, *ED* emergency department, *EDLOS* emergency department length of stay, *SBP* systolic blood pressure, *DBP* diastolic blood pressure, *RR* respiratory rate, *BT* body temperature, *RLQ* right lower quadrant, *RUP* right upper quadrant, *ANC* absolute neutrophil count

**Table 5:** Multivariate logistic regression analysis of factors that affected unfavorable outcomes

Variables	Crude OR (95% CI)	AOR (95% CI)	*p-*value
EDLOS > 4 h	4.05 (2.25–7.3)	2.62 (1.33–5.14)	0.005
**Vital signs**			
DBP < 80 mmHg	2.7 (1.54–4.73)	3.31 (1.71–6.4)	< 0.001
RR ≥ 24 breaths/min	1.59 (0.95–2.66)	2.03(1.07–3.86)	0.031
**Abdominal signs**			
RLQ tenderness	3.34 (1.95–5.73)	3.72 (1.89–7.32)	< 0.001
Abdominal distension	2.12 (1.13–3.97)	2.91 (1.29–6.57)	0.01
Hypoactive bowel sounds	3.73 (1.71–8.16)	2.89 (1.09–7.67)	0.033
Presence of specific abdominal signs	2.1 (1.24–3.54)	2.07 (1.1–3.88)	0.024
**Laboratory results**			
WBC count ≥ 12,000 cells/mm^3^	3.39 (2–5.74)	2.37 (1.22–4.6)	0.011
ANC > 75%	4.4 (2.44–7.93)	2.83 (1.39–5.75)	0.004

*OR* odds ratio, *AOR* adjusted odds ratio, *EDLOS* emergency department length of stay, *DBP* diastolic blood pressure, *RR* respiratory rate, *RLQ* right lower quadrant, *WBC* white blood cell, *ANC* absolute neutrophil count

### Unfavorable outcomes and the mortality group

Significant variables associated with in-hospital mortality in the unfavorable outcome group were the presence of shock, mechanical ventilation used, need for emergency surgery, and occurrence of IHCA (Table 6). The emergency surgical procedures performed included appendectomy (58.5%, 38 patients), exploratory laparotomy (29.2%, 19 patients), laparoscopy (7.7%, 5 patients), and cholecystectomy (4.6%, 3 patients). Three patients died after admission and one patient died in the ED. The diagnoses of the deceased patients in the unfavorable outcome group are shown in Table 7.

**Table 6:** Characteristics in the unfavorable outcome and hospital mortality group

	**Survived** **(*n* = 71)**	**Dead** **(*n* = 4)**	**Total** **(*n* = 75)**	***p*-value**
**Shock**	8 (11.3)	4 (100)	12 (16)	< 0.001
**Type of shock**				
• Septic	4 (50)	3 (75)	7 (58.3)	0.576
• Hypovolemic	5 (62.5)	1 (25)	6 (50)	0.545
• Cardiogenic	1 (12.5)	0 (0)	1 (8.3)	1.000
**Place of shock**				0.632
ED	6 (75)	2 (50)	8 (66.7)	
Ward	1 (12.5)	1 (25)	2 (16.7)	
ICU	0 (0)	1 (25)	1 (8.3)	
EDOU	1 (12.5)	0 (0)	1 (8.3)	
**Invasive procedure performed**				
• CVC insertion	7 (58.3)	4 (100)	11 (68.8)	0.245
• Mechanical ventilation	2 (16.7)	4 (100)	6 (37.5)	0.008
**Emergency surgery**	64 (90.1)	1 (25)	65 (86.7)	0.007
• Exploratory laparotomy	18 (25.3)	1 (25)	19 (25.3)	
• Appendectomy	38 (58.3)	0	38 (58.3)	
• Laparoscopy	5 (7.7)	0	5 (7.7)	
• Cholecystectomy	3 (4.6)	0	3 (4.6)	
**Postoperative complication**	5 (7)	1 (25)	6 (8)	0.289
**In-hospital cardiac arrest**	0 (0)	2 (50)	2 (2.7)	0.002

Data are presented as *n* (%)

*ED* emergency department, *ICU* intensive care unit, *EDOU* emergency department observation unit, *CVC*, central venous catheter

**Table 7:** Diagnoses of the deceased patients in the unfavorable outcome group

No	Profile	Diagnosis	Adverse events/outcome	Surgical procedure	EDLOS	Hospital mortality
1	52 YOM Liver cirrhosis, AIHA on prednisolone	Acute cholecystitis, secondary peritonitis	Septic shock, CVC insertion, mechanical ventilation, cardiac arrest	No	8 h 33 min	Dead at the ED
2	83 YOM Ischemic heart disease	Acute cholangitis (gallstone), splenic infarction	Septic shock, CVC insertion, mechanical ventilation	ERCP	5 h 15 min	5 days
3	83 YOF CKD, COPD	Colitis, diverticular disease	Septic shock, CVC insertion, mechanical ventilation, cardiac arrest	No	6 h 10 min	5 days
4	79 YOM Ischemic stroke	Ruptured infrarenal AAA	Hypovolemic shock, CVC insertion, mechanical ventilation, post-op complication	Open abdominal aortic repair	1 h 15 min	26 days

*EDLOS* emergency department length of stay, *YOM* year-old male, *AIHA* autoimmune hemolytic anemia, *YOF* year-old female, *CKD* chronic kidney disease, *COPD* chronic obstructive pulmonary disease, *AAA* abdominal aortic aneurysm, *CVC* central venous catheter, *ERCP* endoscopic retrograde cholangiopancreatography

## Discussion

There are various definitions of unfavorable outcome related to acute abdominal pain. Most studies focused on the risk associated with surgical intervention or post-operative complications that included surgical site infection, severe sepsis, hospital readmission, admission to the ICU, prolonged hospital stay, and increased hospital mortality [14, 15].

The statistically significant factors (i.e., clinical signs) that were associated with the occurrence of unfavorable outcomes in the multivariate logistic regression analysis were DBP < 80 mmHg, tachypnea (RR ≥ 24 breaths/min), RLQ tenderness, abdominal distension, hypoactive bowel sounds, presence of specific abdominal signs (i.e., Murphy’s sign, Rovsing’s sign, psoas sign), leukocytosis (WBC count ≥ 12,000 cells/mm^3^), ANC > 75%, and EDLOS longer than 4 h.

In our analysis, the vital sign parameters measured at the triage area that were identified as significant factors for the occurrence of unfavorable outcomes in both the univariate and multivariate logistic regression analyses were DBP < 80 mmHg with an adjusted odds ratio (AOR) of 3.31 and RR ≥ 24 breaths/min with an AOR of 2.03. This is in accordance with the result of Barfod’s study. They found that abnormal RR, oxygen saturation (SpO_2_), and Glasgow Coma Scale score were significant risk factors associated with adverse outcome and in-hospital mortality [5]. The study population in this cohort, with abdominal pain as the chief complaint accounted for 20.1% of the study patients and had an in-hospital mortality rate of 3.1%. Blood pressure < 80 mmHg and RR of 26–30 breaths/min had AOR values of 3.87 and 1.89, respectively, but in-hospital mortality was not statistically significant [5]. Increased in-hospital mortality was associated with abnormal vital signs or the presence of hypotensive shock during the ED visit. One large observational multicenter study conducted in adult patients visiting the ED reported that 14% of the study population presented with abdominal pain. The study concluded that the in-hospital patient mortality rate increased gradually with worsening SBP and DBP values. SBP values of 81–100 mmHg and 0–80 mmHg had AORs of 2.62 and 4.07 for mortality, respectively, whereas DBP values of 61–80 mmHg and 0–60 mmHg had AORs of 1.23 and 2.12 for mortality, respectively. However, the study did not report clear cut-off points for SBP, DBP, SpO_2_, or heart rate and did not provide AORs for mortality. The AOR for RR gradually increased between 10 and 19 breaths/min with a substantial increase in mortality at 22 breaths/min [16]. Tringali et al.’s study reported that DBP values below 70 mmHg were associated with increased all-cause mortality in patients aged 45 years or older who encountered outpatient care [17]. Most studies used DBP ≤ 60 mmHg to indicate an impending serious adverse event; however, at this low level, it may be significantly late to detect the abnormality, which may lead to delayed treatment [4, 5, 17]. A previous study explored the predictors of poor outcomes in geriatric patients with acute abdominal pain. According to the study, hypotension, abnormal abdominal radiography findings, leukocytosis, abnormal bowel sounds, and advanced age were the independent predictors of unfavorable outcomes [18].

Ancillary studies should be used only as adjunct information for the clinician's diagnosis based on the clinical symptoms and signs. A diagnostic test resulted in changing the diagnosis in 37% of patients and changing the disposition in 41% of patients in a small prospective trial that evaluated diagnostic testing for nontraumatic abdominal pain in the ED [19]. A complete blood count helps to determine the diagnosis but it is nonspecific and rarely leads to therapy modification. Up to 80% of patients with acute appendicitis may have a high WBC count, but 70% of patients with other causes of RLQ abdominal pain also have an elevated WBC count [20]. However, evidence from a previous study showed that leukocytosis and relative lymphopenia were the only variables meaningfully associated with the presence of a major pathology on computed tomography, and the coexistence of these two anomalies may be sufficient to justify abdominal computed tomography [21]. In accordance with our findings, leukocytosis (WBC count ≥ 12,000 cells/mm^3^) and an ANC > 75% were associated with unfavorable outcomes with AOR values of 2.37 and 2.83, respectively. One of the unfavorable outcome indicators in the present study was the need for emergency surgery, which may indicate to a significant intra-abdominal pathology.

A combination of abdominal signs and presenting symptoms of a patient provides fundamental clinical information clues to establish a diagnosis. The present study explored several physical signs associated with unfavorable outcomes, including RLQ tenderness, abdominal distension, hypoactive bowel sounds, and presence of specific abdominal signs (Murphy’s sign, Rovsing’s sign, psoas sign). Murphy’s sign for acute cholecystitis and Rovsing’s sign and the psoas sign for acute appendicitis are specific abdominal signs that increase the likelihood of an intra-abdominal pathology, which can lead to a precise diagnosis with a wide range of sensitivity and specificity values [22]. From the results of the current study, 48% (36/75) of the patients in the unfavorable outcome group had acute appendicitis. Thus, RLQ tenderness and certain abdominal signs (i.e., Rovsing’s and psoas signs) were associated with unfavorable outcome. Since the abdomen of patients with severe peritonitis is often distended with hypoactive to absent bowel sounds [23], these clinical presentations were significant factors in predicting unfavorable outcomes in our study. One cross-sectional hospital-based longitudinal case series analysis of patients admitted and operated on for acute abdominal pain found that the most frequent signs observed were abdominal tenderness (78.3%), abdominal distension (67.8%), and abnormal bowel sounds (49.7%). They also identified less common abdominal signs, which included guarding (39.2%), abdominal mass (24.5%), positive rectal exam (36.4%), and positive vaginal exam (10.5%), which were found to be significantly associated with adverse outcomes [3]. The results of that study were similar to our study in that generalized/localized abdominal rigidity or abdominal guarding in the univariate logistic regression analysis indicated significantly high odds ratios of 39.68 (95% CI: 10.49, 150.1) and 22.31 (95% CI: 7.86, 63.3), respectively. However, these parameters were not identified in the multivariate logistic regression analysis. The study population in the referenced study was different from that in our study because they included patients of all age groups, but we included only adult patients.

In the present study, four patients in the unfavorable outcome group died within 28 days after admission (Table 7). Three of them had an EDLOS longer than 4 h due to the severity of septic shock and the need for critical care interventions. The patient who died at the ED at 8 h 33 min presented with liver cirrhosis and autoimmune hemolytic anemia and was taking immunosuppressive medication that altered his immune function and defense mechanism against infection [24]. The ability to effectively manage and treat critically ill patients in the ED decreases with overcrowding. EDLOS is a crucial metric for tracking the effectiveness of ED management and has a direct effect on ED overcrowding. Hospital admission rates, 10-day mortality, and dissatisfaction have been associated with longer EDLOS durations [25, 26]. A previous study conducted in our institute reported on the significant factors associated with EDLOS ≥ 4 h in patients who presented with abdominal pain in the ED. After performing multivariate logistic regression analysis, age, rounds of blood testing, interdepartmental consultation, and the need for ultrasonography were associated with an EDLOS ≥ 4 h. We also demonstrated that mortality occurred in a small number of patients who experienced an extended EDLOS. The patients were diagnosed psoas abscess, ruptured hepatoma, acute pancreatitis, and intestinal obstruction with EDLOS times of 12 h 14 min, 4 h 10 min, 5 h 40 min, and 5 h, respectively [26]. However, our previous study did not explore the possible association between EDLOS and unfavorable outcomes.

### Limitations

This study has several limitations. First, it was retrospective in nature and conducted in a single ED. Second, the patients were randomly selected using a computerized procedure; therefore, some characteristics may not have been presented, especially in the unfavorable outcome group. Third, we did not perform a subgroup analysis of patients who underwent emergency surgery, which may have revealed more specific information.

## Conclusions

The present study revealed that significant clinical signs associated with unfavorable outcome were DBP < 80 mmHg, tachypnea (RR ≥ 24 breaths/min), RLQ tenderness, abdominal distension, hypoactive bowel sounds, and presence of specific abdominal signs. Moreover, the associated laboratory results identified in this study were leukocytosis (WBC count ≥ 12,000 cells/mm^3^) and ANC > 75%. Finally, patients with abdominal pain visiting the ED who had an EDLOS > 4 h were associated with unfavorable outcome.

## Supplementary Information

**Additional file 1: Supplementary 1.** Comparison of abdominal imaging, investigationresults, and outcomes.

## Data Availability

Retrospective data used to support the findings of this study are available from the corresponding author upon request.

## Abbreviations

AAA: Abdominal aortic aneurysm
AIHA: Autoimmune hemolytic anemia
ANC: Absolute neutrophil count
AOR: Adjusted odds ratio
AUROC: Area under receiver operating curve
BT: Body temperature
CI: Confidence interval
CVC: Central venous catheter
DBP: Diastolic blood pressure
ED: Emergency department
EDLOS: Emergency department length of stay
EDOU: Emergency department observation unit
ESI: Emergency severity index
ICU: Intensive care unit
IHCA: In-hospital cardiac arrest
LOS: Length of stay
LR −: Negative likelihood ratio
LR +: Positive likelihood ratio
NPV: Negative predictive value
OR: Odds ratio
PPV: Positive predictive value
RLQ: Right lower quadrant
ROC: Receiver operating curve
RR: Respiratory rate
RUQ: Right upper quadrant
SBP: Systolic blood pressure
SpO_2_: Oxygen saturation
WBC: White blood cell
YOF: Year-old female
YOM: Year-old male
