# Supplementary material for: Factors associated with unfavorable outcomes in patients with acute abdominal pain visiting the emergency department
Source: BMC Emerg Med. 2022 Dec 6;22:195. doi: 10.1186/s12873-022-00761-y (PMC9727909; doi:10.1186/s12873-022-00761-y)
Supplement: Supplementary file 1 — Additional file 1: Supplementary 1. Comparison of abdominal imaging, investigationresults, and outcomes. [file 12873_2022_761_MOESM1_ESM.docx]

**Supplementary 1** Comparison of abdominal imaging, investigation results, and outcomes

|  | Unfavorable outcome (n=75) | | Favorable outcome (n=876) | | Total | *p-*value | |
| --- | --- | --- | --- | --- | --- | --- | --- |
| Acute abdomen series | 36 (48) | | 145 (16.6) | | 181 (19) | < 0.001 | |
| Air under diaphragm | 3 (8.3) | | 0 (0) | | 3 (1.6) | 0.007 | |
| Multiple air-fluid levels | 9 (25) | | 9 (6) | | 18 (9.6) | 0.002 | |
| Small bowel dilatation | 10 (27.8) | | 12 (7.9) | | 22 (11.8) | 0.003 | |
| Large bowel dilatation | 3 (8.3) | | 8 (5.3) | | 11 (5.9) | 0.445 | |
| Non-specific finding | 15 (41.7) | | 114 (75.5) | | 129 (69) | < 0.001 | |
| Other findings | 5 (13.9) | | 21 (13.9) | | 26 (13.9) | 1.000 | |
| Ultrasonography | 24 (32) | | 23 (2.6) | | 47 (4.9) | < 0.001 | |
| Localized pelvic fluid | 2 (8.3) | | 0 (0) | | 2 (4.3) | 0.489 | |
| Acute calculus cholecystitis | 1 (4.2) | | 2 (8.7) | | 3 (6.4) | 0.609 | |
| Acute acalculous cholecystitis | 0 (0) | | 1 (4.3) | | 1 (2.1) | 0.489 | |
| Free peritoneal fluid | 4 (16.7) | | 2 (8.7) | | 6 (12.8) | 0.666 | |
| Others | 22 (91.7) | | 19 (82.6) | | 41 (87.2) | 0.416 | |
| Computed tomography | 30 (40) | | 24 (2.7) | | 54 (5.7) | < 0.001 | |
| CT results |  | |  | |  | 0.082 | |
| Positive | 30 (100) | | 21 (87.5) | | 51 (94.4) |  | |
| Negative | 0 (0) | | 3 (12.5) | | 3 (5.6) |  | |
| Other imaging | 28 (37.3) | | 115 (13.1) | | 143 (15) | < 0.001 | |
| ED to US time (hours), mean (SD) | 4.3 (1.9) | | 5.3 (2.7) | | 4.8 (2.4) | 0.129 | |
| ED to CT time (hours), median (IQR) | 5.18 (4.23,17.7) | | 5.4 (4.3,11) | | 5.3 (4.23,7.2) | 0.589 | |
| CBC | 74 (98.7) | 297 (33.9) | | 371 (39) | | | < 0.001 |
| WBC median (IQR), cells/mm^3^ | 13,475 (10,530,15865) | 10,182 (7,900,12,935) | | 10755 (8,197.5,13,797.5) | | | < 0.001 |
| PMN median (IQR), (%) | 81.2 (76,86.5) | 72 (62.8,81.8) | | 75 (64.3,83) | | | < 0.001 |
| Lymphocyte, median (IQR), (%) | 12.3 (8,17.1) | 19.4 (12,28.4) | | 17.7 (11.2,26.7) | | | < 0.001 |
| Mono, median (IQR), (%) | 4.1 (2.9,5.2) | 5.2 (4.1,7) | | 5 (4,6.8) | | | < 0.001 |
| Hb, median (IQR), g/dL | 12.9 (11.6,14.1) | 12.9 (11.9,13.7) | | 12.9 (11.8,13.7) | | | 0.995 |
| Platelet median (IQR), cells/mm^3^ | 236,000 (209,250,286500) | 257,500 (216,750,326,750) | | 255,500 (214,000,323,250) | | | 0.046 |
| Arterial blood gas, n (%) | 12 (16) | 9 (1) | | 21 (2.2) | | | < 0.001 |
| Lactate, median (IQR), mmol/L | 3 (2.5,6.8) | 1.1 (0.9,1.7) | | 2.5 (1.3,3.6) | | | 0.004 |
| Blood chemistry, n (%) | 70 (93.3) | 257 (29.3) | | 327 (34.4) | | | < 0.001 |
| LFT | 26 (38.2) | 64 (25.5) | | 90 (28.2) | | | 0.055 |
| TP, median (IQR) | 7.1 (6.6,7.5) | 7.4 (7,7.7) | | 7.3 (6.9,7.6) | | | 0.025 |
| ALB, median (IQR) | 3.8 (2.9,4.3) | 4.3 (4,4.6) | | 4.2 (3.8,4.5) | | | 0.005 |
| Urinalysis |  |  | |  | | | < 0.001 |
| Positive | 11 (17.2) | 164 (41.1) | | 175 (37.8) | | |  |
| Negative | 53 (82.8) | 235 (58.9) | | 288 (62.2) | | |  |
| Coagulation study |  |  | |  | | | 0.588 |
| Normal | 49 (96.1) | 61 (98.4) | | 110 (97.3) | | |  |
| Prolonged | 2 (3.9) | 1 (1.6) | | 3 (2.7) | | |  |
| Hemoculture result |  |  | |  | | | 0.489 |
| Positive | 5 (26.3) | 7 (16.7) | | 12 (19.7) | | |  |
| Negative | 14 (73.7) | 35 (83.3) | | 49 (80.3) | | |  |
| Other culture |  |  | |  | | | 0.401 |
| Positive | 8 (53.3) | 28 (70) | | 36 (65.5) | | |  |
| Negative | 7 (46.7) | 12 (30) | | 19 (34.5) | | |  |

Data are presented as *n* (%) unless otherwise indicated.

*CT* computed tomography, *ED* emergency department, *US* ultrasonography, *CBC* complete blood count, *WBC* white blood cell count, *PMN* polymorphonuclear cells, *Hb* hemoglobin, *IQR* interquartile range, *LFT* liver function test, *TP* total protein, *ALB* albumin
